# Ceramic liner fracture in ceramic on ceramic Total hip arthoplasty: A case report

**DOI:** 10.1016/j.ijscr.2019.10.079

**Published:** 2019-11-05

**Authors:** Eknath D. Pawar, Amit Kumar Yadav, Arohi Sharma, Abhishek Harsoor

**Affiliations:** Grant Govt Medical College & Sir JJ Group of Hospitals, Mumbai, India

**Keywords:** Ceramic on ceramic, Total hip arthroplasty, Ceramic on polyethylene

## Abstract

**Introduction:**

Revision rates of total hip arthoplasty have decreased after introducing total hip arthroplasty using ceramic component due to reduce wear and osteolysis.

**Case report:**

29 year old male case of bilateral hip avascular necrosis operated for bilateral hip THA presented 30 months after with complaint of squeaking, x-ray showing left ceramic liner fracture without any history of trauma. Revision of ceramic liner and head with ceramic on polyethylene was done.

**Discussion and conclusion:**

Ceramic on ceramic articulation has the lowest wear rate among various articulations. However, there is a concern about ceramic implant fracture; therefore ceramic with polyethylene bearing could be a good choice.

## Highlights

• A case of ceramic linear fracture without trauma.
• Ceramic with polyethylene bearing could be a good choice after ceramic on ceramic liner fracture.

## 1 Introduction

Revision rates of Total hip arthroplasty (THA) have decreased after introducing THA using ceramic component, since ceramic components could reduce components wear and osteolysis owning to wear particles from metal or polyethylene [1]. Ceramic-on-ceramic articulation has the lowest wear rate among various articulation [2]. However, Ceramic articulations have not been without their problems, specifically squeaking and implant fracture. The fracture of a ceramic component is a rare but potentially serious event. Thus, ceramic-on-polyethylene articulation is gradually spotlighted to reduce impact force between hard ceramic materials, as well as lower wear rates of polyethylene liner than metal-on-polyethylene articulation [3].

We report a case report of ceramic liner failure 30 months after THA managed in our tertiary hospital. This work has been reported in line with the SCARE 2018 criteria [3].

## 2 Case report

**Fig. 1:**
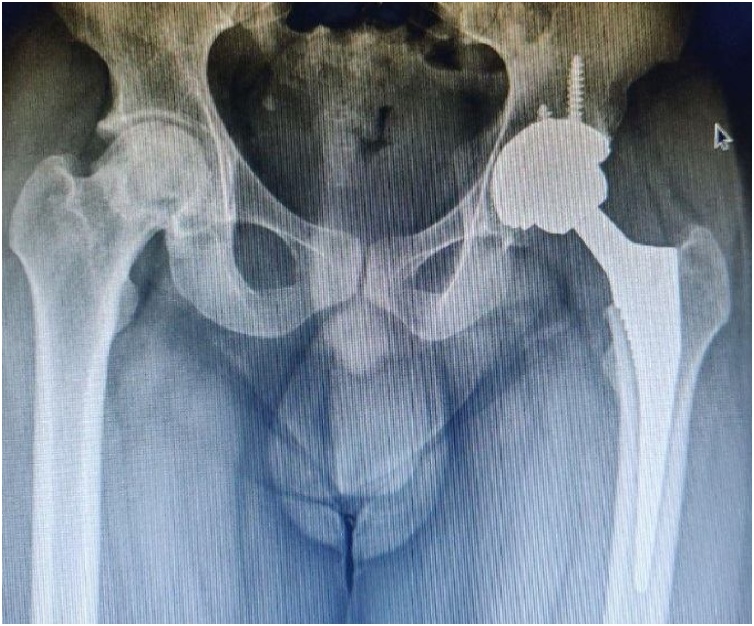
Showing postoperative AP radiograph a normal seated acetabular component and liner.

A 29 year old male a case of bilateral hip avascular necrosis underwent a bilateral primary cementless THA in year 2016. The former left arthroplasty include the (Corail HA coated standard no collar ks size 9 stem, with a 50 mm pinnacle (Depuy synthesis) acetabular shell). A Biolox Delta ceramic femoral head (32 mm + 9.0) and 32 mm neutral liner were used as bearing surfaces. The inclination of acetabular cup was 42^0^ and anteversion was 12 ^0^. Fig. 1 demonstrate the post-operative anteroposterior radiograph.

**Fig. 2:**
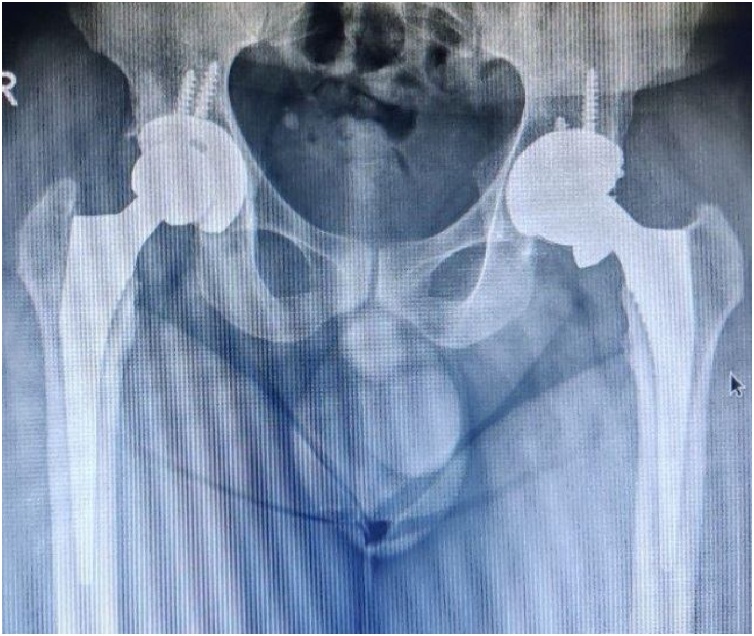
Shows a left side ceramic liner fracture.

After 30 month he presented with squeak in her hip. He had no problem in gait and no history of trauma and squatting. There was no evidence of infection around left hip. Fig. 2 showing the anteriorposterior radiograph.

**Fig. 3:**
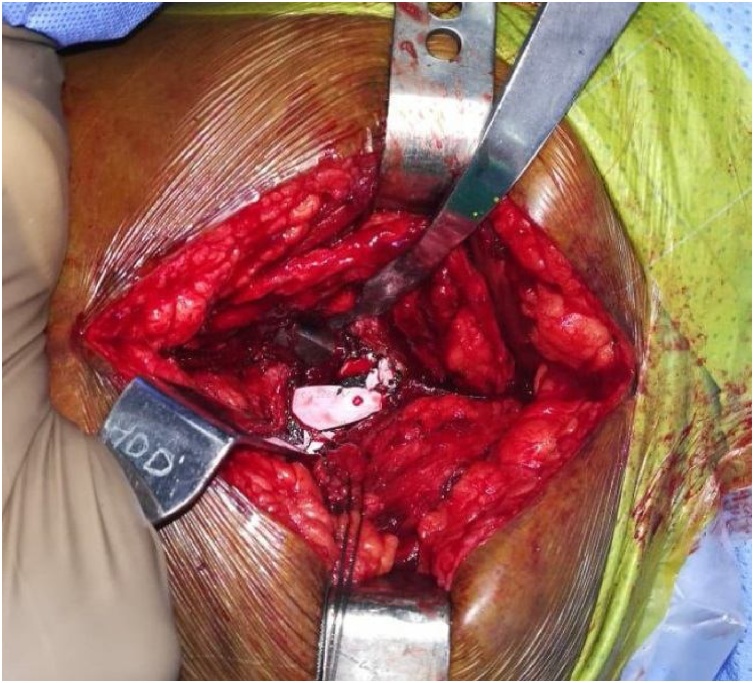
Intraoperative photographs shows Fractured Ceramic liner.

Revision was done through Posterolateral approach similar to previous operation. After capsulectomy, various sizes of ceramic particles were observed (Fig. 3). Intraoperatively, ceramic fragments were meticulously removed, and extensive capsulectomy was performed. The ceramic liner was exchanged for a polyethylene liner and a new ceramic head (Polyethylene liner 32 mm + 4 mm 10^0^, Biolox delta ceramic 32 mm + 5.0). Thus, femoral stem and acetabular cup was well fit so retain. After the removal of all components, massive irrigation and extensive synovectomy were done to remove microscopic ceramic fragments.

**Fig. 4:**
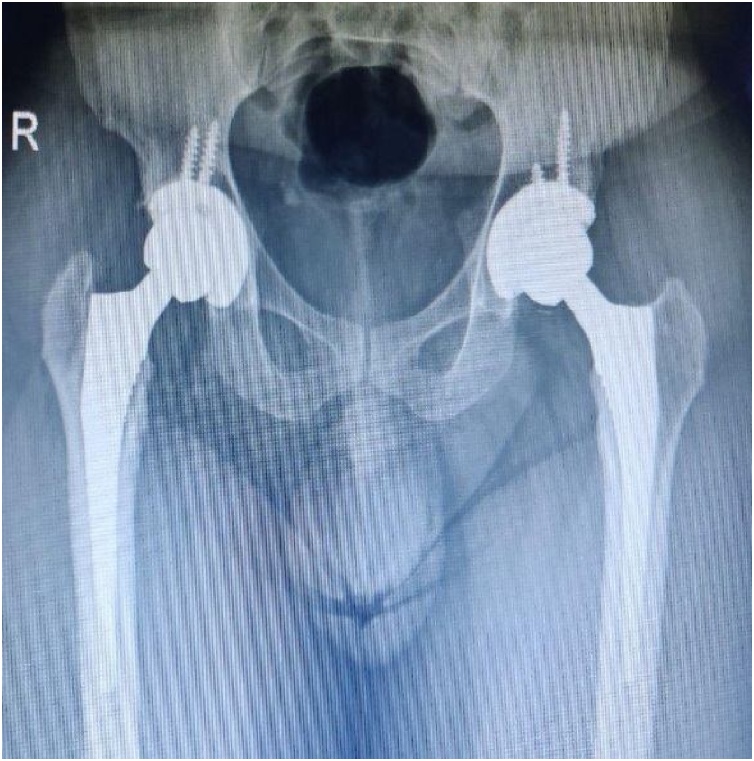
Radiograph after revision of ceramic Head and liner.

At his 12 months follow-up, he could return to her previous level of activity, and had Harris hip score of 93. In radiographs, the implant showed stable fixation without sign of osteolysis or loosening (Fig. 4).

## 3 Discussion

Ceramic materials have been used for THA with better clinical results, compared with conventional metal-on-polyethylene articulation [1]. Ceramic on ceramic articulation has the lowest wear rate among various articulations. awHowever, there is a concern about ceramic head fracture, and squeaking therefore, ceramic-on-polyethylene combinations are becoming more popular than ceramic on ceramic. Several clinical studies [4] found that ceramic-on-polyethylene articulations have lower wear rate than those of metal-on-polyethylene articulation. In a study group of 1382 Ceramic on ceramic hips, there was only 1 fracture of an acetabular insert (0.07 %) [5]. The authors of that study estimated the risk of fracture over the lifetime of these implants to be closer to 1 in 2000–3000 for femoral heads (0.05 %–0.03 %) and 1 in 6000–8000 for the acetabular insert (0.017 %–0.013 %). Fractures of ceramic components tend to occur within the first few years after implantation. Data collected by Biolox manufacturer, CeramTec AG, on nearly 2 million implants indicated that 50 % of ceramic component fractures occur within the first 12 months, 70 % within 24 months, and 83 % within 36 months [6]. It is very rare but catastrophic event which requires revision operation. Rates of use of Ceramic on ceramic bearings have recently declined owing in part to their brittle nature and four fold increased risk of implant fracture over Ceramic on polyethylene bearings.

Revision surgery for fractured ceramic component should be performed urgently in order to reduce the risk of damage to the components from ceramic particles and surgery should always include an extensive synovectomy and thorough irrigation; complete elimination of ceramic debris has been shown to increase the survivorship of the new articulation [7]. There remains significant debate as to the most appropriate bearing couple for revision after fractured ceramic component. Although there are concerns about accelerated polyethylene wear in such a revision, both Ceramic on ceramic and ceramic on polyethylene couples are considered reasonable selections as the use of ceramic heads exhibit increased Scratch resistance and could reduce the risk of third-body wear.

## 4 Conclusion

We conclude that Ceramic on ceramic should be used in THA especially in young patients due to its long-term survival rates and low rates of revision surgery but the best option bearing surface to change after a fracture in ceramic on ceramic is not defined yet, and we believe that ceramic-polyethylene bearing with an additional extended capsulectomy could be a good choice

## Funding

None.

## Ethical approval

This is case report study, no ethical approval were needed. In the other hand, all patient had been informed and gave their consent regarding this publication.

## Consent

Written informed consent was obtained from the patient for publication of this case report and accompanying images. A copy of the written consent is available for review by the Editor-in-Chief of this journal on request.

## Author’s contribution

Dr eknath Pawar- contributed in performing the surgical procedure.

Dr amit kumar yadav- contributed in Evaluation and post-operative management of the case along with surgical assistance, writing the paper.

Dr Arohi sharma – contributed in surgical procedure.

Dr abhishek harsoor – contributed in data collection.

## Registration of research studies

This is a case report. Hence, it is not registered in the clinical trial registry.

## Guarantor

Dr amit kumar Yadav.

## Provenance and peer review

Not commissioned externally peer reviewed.

## Declaration of Competing Interest

None.
